# Bio-Insecticide of *Thymus vulgaris* and *Ocimum basilicum* Extract from Cell Suspensions and Their Inhibitory Effect against Serine, Cysteine, and Metalloproteinases of the Red Palm Weevil (*Rhynchophorus ferrugineus*)

**DOI:** 10.3390/insects12050405

**Published:** 2021-04-30

**Authors:** Hossam Moustafa Darrag, Mohammed Refdan Alhajhoj, Hany Ezzat Khalil

**Affiliations:** 1 Research and Training Station, King Faisal University, Al-Ahsa 31982, Saudi Arabia; 2 Pesticide Chemistry and Technology Department, Faculty of Agriculture, Alexandria University, Alexandria 21545, Egypt; 3 Arid Land Agriculture Department, College of Agricultural and Food Sciences, King Faisal University, Al-Ahsa 31982, Saudi Arabia; malhajhoj@kfu.edu.sa; 4 Department of Pharmaceutical Sciences, College of Clinical Pharmacy, King Faisal University, Al-Ahsa 31982, Saudi Arabia; heahmed@kfu.edu.sa; 5 Department of Pharmacognosy, Faculty of Pharmacy, Minia University, Minia 61519, Egypt

**Keywords:** *Thymus vulgaris*, *Ocimum basilicum*, proteolytic enzymes, serine, cysteine, metalloproteinase, antifeedant, *Verticillium dahliae*

## Abstract

**Simple Summary:**

Most thyme and basil species are characterized by a great chemical diversity. Uses have been developed through centuries in foods, as a source of essential oil, flavors, and antioxidants. The main aim of this study was to produce volatile metabolites using cell suspensions of *Thymus vulgaris* and *Ocimum basilicum* from the Al-Ahsa area in the sub-continental region. We surveyed the antifeedant activity of extracted volatile metabolites and the inhibition of extracts against total proteolytic enzyme activity from the red palm weevil *Rhynchophorus ferrugineus* (Oliver), (Coleoptera: Curculionidae). *Thymus vulgaris* was the most active extract, characterized not only by feeding inhibition but also by a growing deterrence on *R. ferrugineus* larvae. The *O. basilicum* extract also showed a lower range of biological activity; nevertheless, there was potent insecticidal activity. The high insecticidal activity of the *T. vulgaris* extract could be attributed to the high diversity of its volatile constituents. One of the merits of the current approach is that the outcomes are applicable and have the environmental goal of producing ecofriendly biopesticides.

**Abstract:**

The current study was designed to investigate the insecticide role of volatile constituents produced from cell suspensions of *T. vulgaris* and *O. basilicum* against *R. ferrugineus*. Constituents were extracted from cell suspension after 40 days. Growth kinetics were measured with an inoculation of *Verticillium dahliae* and identified by GC-MS. Total volatile phenolic constituents were measured. Insecticidal activity against *R. ferrugineus* (adult) and proteolytic enzyme activity in larvae were assessed. GC-MS showed that the *T. vulgaris* extract has higher amounts of thymol, *p*-cymene, γ-terpinene, β-caryophyllene, and linalool in comparison to the *O. basilicum* extract, which is rich in estragole, β-terpineol, (*E*)-β-ocimene, 1,8-cineole, germacrene D, and eugenol. The *T. vulgaris* extract showed an LC_50_ of 1032 µg/mL, followed by *O. basilicum* with an LC_50_ of 1246 µg/mL. The IC_50_ values against the total proteases were 110.8 and 119.4 µg/mL for *T.* *vulgaris* and *O.* *basilicum*, respectively. The IC_50_ for the trypsin-like serine proteinase assessment was 81.6 and 91 µg/mL for *T.* *vulgaris* and *O.* *basilicum*, respectively. Cysteine, chymotrypsin, and metalloproteinase assessment showed an IC_50_ above 5000 µg/mL for both extracts. The study is proposed as a potential approach to use *T. vulgaris* and *O. basilicum* extract as a bio-insecticide against *R. ferrugineus* using an accessible and efficient cell suspension technique.

## 1. Introduction

Date palm (*Phoenix dactylifera* L.) is one of the most important economic crops. Date palm is commonly attacked by a variety of pests during its growth season [1]. Many investigators have indicated that infested palms usually serve as a source pocket for further spread of infestation, which, for its successful control, depends upon the early detection of infested trees to be immediately treated [2]. Methods of its control were mainly preventive and mechanical, with the early trials focusing on the utilization of chemical insecticides [3,4,5,6]. However, legislators and investigators intend to prevent the use of these chemicals for their negative impacts on both the environment and all other natural resources [4,5,6]. When the red palm weevil*, Rhynchophorus ferrugineus* Olivier (Coleoptera, Curculionidae), spreads in urban areas, it becomes hard to eliminate its outbreaks, which accelerates the extent of infestation and continues to threaten palm trees in outlying wilderness areas and agricultural landscapes nearby; therefore, quarantine measures are essential during live date palm transfer [7].

The red palm weevil *R. ferrugineus* (Oliver) (Coleoptera: Curculionidae) is a dangerous polyphagous insect. In addition, it is one of the most important pests of numerous palm species; it is reported to attack more than 21 palm species worldwide, including date palm [8], which leads to losses in crop production. The larvae are considered one of the most difficult and most harmful stages, as they spread and penetrate the palm trees significantly. They cause the death and deformation of palm fronds due to their feeding on the apical meristem, significantly devastating palm trees [9,10]. Once palm trees are infested, it becomes easily infected with many insects, fungal diseases, and other pests through the tunnels caused by palm weevil larvae [2,7,11,12].

The species *R. ferrugineus* is found and spread across the Middle East (arid regions) and the Mediterranean region, which includes North Africa and Europe as well as tropical regions, and it is the most destructive of the ten species of genus *Rhynchophorus*, which is generally found and distributed in the pan-tropical areas [13,14,15,16,17].

Numerous biologically active constituents have been isolated from natural resources (including volatile oils, fixed oils, and other plant constituents) that can be used against insect species [18]. The larvicidal effect may be attributed to the various chemical components that have been isolated, e.g., terpenoids, flavonoids, alkaloids, sterols, and others [19]. Some of natural isolated constituents showed larvicidal effects, such as filiferol, which was significantly appreciable with respect to *R. ferrugineus* [20]. Extracts of *Justicia brandegeana* have a potent effect as a biocontrol agent against the red palm weevil. They showed a possible effect on enzymatic bioactivity and demonstrated the effectiveness of chitinase and a disturbance effect on the enzymatic system, protein, and DNA damage [8]. Recent reports demonstrated the effect of the latex of *Calotrapos gigantean* as serine protease inhibitors (SPIs) that can work as an insecticide of *R. ferrugineus* in the midgut [21]. Reports have substantiated the efficacy of protease inhibitors against diverse biotic factors and the associated protecting properties in crops, representing potential environmentally friendly agrochemicals [22]. Monoterpene derivatives showed pesticidal properties, rendering them as good leads for the development of safe and ecofriendly agents [23]. Several secondary metabolites could also be used to monitor the red palm weevil, with the prospect of using 1-octen-3-ol, geraniol, and α-pinene for RPW population management based on field and laboratory data [24]. In particular, α-pinene, alone or in combination with methyl salicylate, evidenced pheromone-disrupting properties [25]. Moreover, coumarin silenced the genes involved in the *R. ferrugineus* detoxification mechanism and could be used as a natural controlling agent in the future [26], and picrotoxin could be used as a biopesticide for the control of red palm weevil infestations [27].

In addition, innumerable genera such as *Thymus* and *Ocimum* of the Lamiaceae family produce several separate classes of secondary metabolites, such as flavonoids, terpenoids, phenols, and alkaloids, which have applications and biological activities, including anti-inflammatory, antioxidant, and anti-bacterial activities [28,29,30,31,32,33]. Leading components such as monoterpenes, sesquiterpenes, derivatives of phenylpropanoids, and flavonoids were identified in many members of the family Lamiaceae, including *T. vulgaris* and *O. basilicum* [34,35], and could be assessed as bio-insecticides. To the best of our knowledge, no one has characterized *T. vulgaris* or *O. basilicum* of Saudi origin, especially from the eastern province, the Al-Ahsa region, as bio-insecticides against the red palm weevil. Consequently, no reports, such as a detailed chemical composition using cell suspension of volatile metabolites from *T. vulgaris* and *O. basilicum* from the Al-Ahsa area (Miqdaam, Al-Shoaba, and Almterfi areas), ethnic to the sub continental region, are available. 

In this context, tissue cultures and plant cells hold controlled secondary metabolite production. Productivity and current yield cannot reach the bioprocess targets of plant cells to increase secondary metabolite production. The opportunities in plant-cell-based processes, new directions, and recent advances are being critically tested. The exact reproduction of plants has been achieved using genotypes and somatic embryogenesis by different explants (meristematic cells) such as embryos, offshoots, and buds. Somatic embryogenesis is more efficient in the process of plant multiplication and can be used for secondary metabolite production. Previous studies have been conducted to optimize plant somatic embryogenesis through the manipulation of culture medium ingredients, including amino acids, auxins, cytokinins, abscisic acid, *N*-phenyl *N*′-1,2,3-thidiazol-5-ylurea (TDZ), sucrose, biotin, thiamine, basal salt formulations, and strong organic additives for direct somatic embryos [36,37]. 

Limited research has been carried out for bioactive compound production from plants in in vitro cultures. Recently, there has been interest in the in vitro production of secondary metabolites from plants using cell suspension cultures. Our study establishes optimization culture conditions for bioactive compound production from plants grown in this region. The work is aimed at developing a bioreactor-scale production of bioactive compounds from cell suspension cultures of plants.

Thus, the present work aims to study the growth kinetics of cell suspensions of *O. basilicum* and *T. vulgaris* to produce biologically active volatile constituents using an easy and clean technique (cell suspension). The composition of the volatile constituents produced from the culture of *O. basilicum* and *T. vulgaris* was analyzed using GC-MS. The insecticidal activity against the larvae and adults of *R. ferrugineus*, including contact-insecticide and antifeedant activities and the inhibition of serine, cysteine, and metalloproteinases of the red palm weevil, is evaluated in vitro. The results are expected to produce an ecofriendly natural bio-insecticide against this pest.

## 2. Materials and Methods

### 2.1. Chemicals and Reagents

Na_2_CO_3_, Na_2_SO_4_, K_2_HPO_4_, KH_2_PO_4_, NaOH and sucrose were provided by Merck Chemical Co., St. Louis, MO, USA. Hydrochloric acid, hypochlorite solution, butanol, acetone, gallic acid, and solvents for extraction, including methanol, *n*-hexane, and NaCl, were provided by Sigma-Aldrich Chemical Co., St. Louis, MO, USA. Reagents for biochemical studies include 2,4-dichloro phenoxy acetic acid (2,4-D), naphthalene acetic acid (NAA), kinetin, indole-3-acetic acid (IAA), indole-3-butyric acid (IBA), phenymethanesulfonyl fluoride (PMSF), dithiothreitol (DTT), azocasien, trichloroacetic acid (TCA), *N*-2-hydroxyelthyl piperazin-*N*′-2-ethanesulphonic acid (HEPES), ferric ammonium sulfate, ethylenediaminetetraacetic acid disodium salt (EDTA), CuSO_4_·5H_2_O, Na-K tartarate, Triton X-100 and Tween 20, and were provided by Sigma-Aldrich Chemical Co., St. Louis, MO, USA. Bovin serium albumin (BSA), Coomassie Brilliant Blue, Folin–Ciocalteu reagent, Na-benzoyl-l-arginine ρ-nitroanilide (BAρNA), *N*-succinyl-ala-alapro-leucine ρ-nitroanilide (SAAPLρNA), Z-Phe-Arg-MNA, mersalyl, leucine ρ-nitroanilide (LpNA), *N*,*p*-tosyl-l-lysine chloromethyl ketone (TLCK), *N*,alpha-tosyl-l-phenylalanine chloromethyl ketone (TPCK), iodoacetic acid, leupeptin and fast garnet reactive solution were provided by Sigma-Aldrich Chemical Co., (St. Louis, MO, USA). Thymol, estragole, *p*-cymene, γ-terpinene, linalool, β-terpineol, ocimene, eugenol, and 1,8-cineole were provided by Merck Chemical Co., (St. Louis, MO, USA). β-Caryophyllene was provided by Sigma-Aldrich Chemical Co., (St. Louis, MO, USA), and germacrene D was provided by Aobious Inc., (9 Blackburn Drive, Gloucester, MA 01930, USA).

### 2.2. Media

Murashige and Skoog (MS) and Linsmaier & Skoog (LS) media (containing 100 mg of myo-Inositol and 0.1 mg of thiamine HCl) were provided by Sigma-Aldrich Chemical Co., (St. Louis, MO, USA). Different media were freshly prepared and autoclaved at 121 °C for 20 min. *Verticillium dahliae* was provided by Microbiology Laboratory, Arid Land Agriculture Department, College of Agricultural and Food Sciences, King Faisal University, Al-Ahsa, Saudi Arabia. Tryptone, yeast extract, and potato dextrose agar (PDA) were purchased from Pronadisa, Madrid, Spain, and Agar from Sigma Chemical Co., (St. Louis, MO, USA). Different media were freshly prepared and autoclaved at 121 °C for 20 min. 

### 2.3. Plant Material

*T. vulgaris* and *O. basilicum* seed plants used in this investigation were from commercial nurseries in the Miqdaam, Al-Shoaba, and Almterfi areas, Al-Ahsa, Saudi Arabia, from February to March. The sterilized seeds (placed in 2% NaOCl for 30 min, followed by rinsing using DW) were placed in the MS medium, pH 5.7, containing agar 0.6% and 3% sucrose, and kept in a climate chamber (26 ± 2 °C, 16 h light conditions) for 8 weeks (seedling 18–20 cm) in the Research and Training Station of King Faisal University, Al-Ahsa, Saudi Arabia.

### 2.4. Callus Initiation of T. Vulgaris and O. basilicum Using Different Plant Growth Regulators with Biotic Elicitor (V. dahliae)

The seeds were delinted, sterilized, and germinated in Petri dishes on sterile blotting paper under 28 ± 2 °C and 30 Einsteins/(m^2^ s) light, and radical emergence was tested. After three days, the explants (hypocotyls, epicotyl, and cotyledonary, with 4~5 mm lengths) of *T. vulgaris* and *O. basilicum* were placed in the MS medium containing (kinetin (0.5 mg/L), 2,4-D (0.1 mg/L), NAA (0.1 mg/L), and IBA (1 mg/L) as plant growth regulators (PGRs) and 3% (*w*/*v*) sucrose according to Muhammed Akif Açıkgöz with modifications [38]). The control treatment was free of any growth regulator, and all treatments were kept in a climate chamber (26 ± 2 °C, 16 h light conditions) for 8 weeks with a subculture every 3 weeks.

*Verticillium dahliae* was used as an initiator (biotic elicitor) to study the growth promotion of callus. Callus was harvested from individual cultures 72 h post-inoculations by vacuum filtration. Finally, calli of *T. vulgaris* and *O. basilicum* were harvested from individual cultures 72 h post-inoculation with *V. dahliae*. Calli were visually evaluated every 5 days for 40 days.

The *V. dahliae* strain was maintained in PDA at 22 °C and subcultured every 5–6 weeks. For experiments, conidia were grown in a potato dextrose liquid medium for 10 days at 22 °C using a rotary shaker (at 240 rpm). Conidia were harvested using centrifugation and washed with 0.1 M K_2_HPO_4_-KH_2_PO_4_ three times, at pH 6.5, and conidia concentration was counted under a microscope using a hemocytometer. A typical experiment was initiated by inoculating 25 µL of conidial suspension [(2–5) × 10^7^ conidia/mL] or sterile water (control), obtained by gravity filtration from a 9-day-old culture (late-log phase), into 8 mL of fresh MS solid medium in a Petri dish. The callus was incubated in the dark at 30 °C for 36 h before adding fungal conidia. 

### 2.5. Initiation of the Cell Suspension of T. vulgaris and O. basilicum and Their Measure Growth Kinetics (Cell Weight)

Calli were initiated and identified using LS media as liquid media for 5–6 weeks. LS media were passed through screens of various mesh sizes. The weight of the cell suspension content was determined (at intervals in 5–40 days) to measure growth kinetics. Liquid culture (200 mL) was filtered and inoculated with 25 µL of conidial suspension [(2–5) × 10^7^ conidia/mL] or sterile water (control). Each inoculated culture was then transferred to 30 Erlenmeyer flasks (500 mL) and adjusted to 250 mL using the LS medium. Seventy-two hours after inoculation, cultures were harvested and analyzed for protein content based on the increase in weight. Subculture (suspension media) was initiated using LS media (100 mL) without gelling agents in conical flasks (250 mL), maintained at 30 ± 2 °C for 16 h of light conditions with a shaker (100 rpm) for 6 weeks, subcultured every 2 weeks, and kept in the climate chamber.

The increase in weight content was determined to measure growth kinetics (at intervals in 5–40 days). Seventy-two hours after inoculation, *V. dahliae* cultures were harvested for protein content detection using increases in the weight (in the callus and the cell suspension). The experiment was carried out with a completely random design (CRD) by means of 3 repetitions in each of the 4 tubes or conical flasks/replicates, with one hypocotyl, epicotyl, and cotyledonary portion per tube. Experiments were repeated twice.

### 2.6. Determination of the Total Volatile Phenolic Content (TVPC) 

Total volatile phenolic constituents were extracted using the hydro distillation method (clevenger apparatus for 3 h) from the callus and cell suspension (after 40 days) and then dried using anhydrous Na_2_SO_4_. Determination of total volatile phenolic content was carried out using the Folin–Ciocalteu method [39]. Distilled water (790 µL) was added to a diluted sample (10 µL), followed by the Folin–Ciocalteu reagent (50 µL), and the mixture was then homogenized using vortexes. Sodium carbonate (20%, *w*/*v*, 150 µL) was added over 1 min, mixed using vortexes again, and the mixture was incubated at room temperature for 120 min in darkness. Total volatile phenolic content was determined via spectroscopy at 750 nm and calculated using the gallic acid standard curve using the serial concentration of gallic acid (1–100 mg of gallic acid per 100 g DW).

### 2.7. Gas Chromatography-Mass Spectrometry Analysis (GC-MS)

Hydro distillate extract from a cell suspension after 40 days was diluted with *n*-hexane (GC grade, 2 μL:1 mL) and injected (1 μL) using an auto-sampler injector (Model Combi Pal, Varian) to the GC–MS (GC, Model CP-3800, Varian, Walnut Creek, CA, USA) linked with a mass spectrometer (MS, Model Saturn 2200, Varian) using a VF-5ms fused silica capillary column (5% phenyl- dimethylpolysiloxane, 30 m × 0.25 mm i.d., film thickness 0.25 μm, Varian, Palo Alto, CA, USA). The electron impact (EI) ionization detector was used with an ionization energy of 70 eV. Helium was a carrier gas (99.99%) with a constant rate (1 mL/min). The injector and mass transfer line temperatures were 240 and 300 °C, respectively. The oven temperature was held at 50 °C for 1 min, raised to 230 °C for 50 min at 30 °C/min, finally raised to 290 °C for 5 min at 10 °C/min and held isothermal for 6 min. The sample split injection ratio was 1/500, with a total time of 54.3 min. The identification of components was based on matching with a mixed standard (*n*-alkanes (C6–C26)) and the Wiley and National Institute of Standards and Technology (NIST) electronic library.

### 2.8. Assessment Contact–Insecticide and Antifeedant Activity of the Extracted Secondary Metabolites against R. ferrugineus 

The serial concentrations of hydro distillate extract from the cell suspension after 40 days (50, 100, 500, 1000, and 5000 µg/mL) were prepared in acetone and then made up volume dried weight (DW) with 0.1% TritonX-100 according to Shukla et al. [40] with modifications. Adult larvae of *R. ferrugineus* were obtained from an insect rearing laboratory in the Date Palm Research Center of Excellence, King Faisal University, Saudi Arabia, and they were reared using sugarcane stem long pieces. Activity of extracts against larvae was measured by keeping larvae at 4–5 °C for 5 min and then using topical application methods. A hand-operated micro-applicator (Burkard Manufacturing Co., Ltd., Hertfordshire, UK) with a 50-ll micro-syringe (MS-N50; Ito Corp., Shizuoka, Japan) was used by an application of 10 µL of previous serial concentrations in dorsum for each larvae (5 larvae for each box and feeding using 10 cm of sugarcane stem long pieces) with 3 replicated for each serial concentration. After topical application, the mortality of the larvae was measured at intervals of 24, 48, 72, and 96 h to calculate LD_50_. Antifeedant activity against adults was measured using 10 cm of sugarcane stem long pieces, split into equal longitudinal halves. Pieces (area ~32.5 cm^2^) were dipped in 10 mL of extract for ten seconds then left to dry in air at room temperature. Each treated piece was placed in a plastic box. One pair (male and female) was replaced in each box. Ten replicates were prepared for each treatment. Observations on feeding were assessed after 24, 48, 72 and 96 h. 

The following equation was used for calculating the total number of pricks on a sugarcane piece, ~32.5 cm^2^):

No. of prick marks = [((P1 + P2)/2) * Z]/4
P1 = maximum numbers of pricks in a window area (2 × 2 cm^2^);
P2 = minimum number of pricks in a window area (2 × 2 cm^2^);
Z = area of sugarcane piece (~32.5 cm^2^) in each replicate.

### 2.9. Determination Effects of T. vulgaris and O. basilicum Extract from Cell Suspension and Pure Compounds on Total Proteolytic Enzymes Activity of R. ferrugineus Larvae

Protein determination was carried out according to the Lowry method [41]. Total proteolytic enzyme activity using azocasein was assessed in the 4th midgut instars larvae homogenate of *R. ferrugineus* according to Olga et al., [42] with modifications. The 4th midgut larvae homogenates of lab strain (10 larvae) were pulled out gently, excised, and washed using a saline solution (0.9% (*w*/*v*) NaCl) repeatedly and then homogenized using 500 µL of an assay buffer. The midgut instar was homogenized in 500 µL of a protease assay buffer [50 mM HEPS (*N*-2-hydroxyelthyl piperazin-*N*′-2-ehtanesulphonic acid), pH 8.0, 5 mM dithiothreitol (DTT) and 0.1% (*v*/*v*) Triton X-100]. The reserve homogenates that were obtained from a previous step were centrifuged at 5000× *g* for 30 min using a Sigma 3k30 cooling centrifuge. The supernatants were used for estimation of the total proteolytic enzyme activity and protein concentration. Ten microliters of supernatant per assay was incubated in a total volume of 60 µL of assay buffer (pH 8) for 20 min at 37 °C before the addition of 200 µL of azocasien (2%, *w/v* in an assay buffer). 

In all cases, enzyme samples of 10 μL, hydro distillate extract from the cell suspension after 40 days, and pure compounds (thymol, estragole, *p*-cymene, γ-terpinene, linalool, β-terpineol, ocimene, eugenol, 1,8-cineole, β-Caryophyllene, and germacrene D) (50, 100, 500, 1000, and 5000 mg/L) were pre-incubated together for 10 min. Substrate was then added to start the reaction (20 min for Leupeptin). The reaction lasted for 180 min at 37 °C and was then stopped using 300 µL of cold 10% (*v*/*v*) trichloroacetic acid (TCA). The reaction mixture was centrifuged at 5000× *g* for 20 min using the Sigma 3k30 cooling centrifuge. Ten microliters of NaOH (10 N) was added to the supernatant, and absorbency at 450 nm was measured using an ELISA plate reader. An assay mixture without an enzyme was used as a blank, the specific activity of total proteolytic enzymes was calculated as OD450. mg^−1^·protein^−1^·h^−1^, and a blank sample was determined without an enzyme solution.

### 2.10. Determination Effects of T. vulgaris and O. basilicum Extract and Pure Compounds from Cell Suspension on Serine Proteinase Specific Activity Assays

Serine proteinase specific activities were assayed, as described by Olga et al. [42] with modifications, using a rapid microplate assay with synthetic substrates in 150 µL reaction mixtures containing a serine protease assay buffer (100 mM Tris-HCl, pH 8.1). Na-benzoyl-l-arginine ρ-nitroanilide (BAρNA) was evaluated for trypsin-like proteinases, *N*-succinyl-ala-alapro-leucine ρ-nitroanilide (SAAPLρNA) for elastase-like proteinases, and *N*-succinyl-ala-ala-pro-phenylalanine ρ-nitroanilide (SAAPFρNA) for chymotrypsin-like proteinases. 

The 4th instar midguts were homogenized with the assay buffer. The homogenates of midguts were centrifuged at 8000× *g* for 30 min using Sigma 3K 30 rotors No. 12158 (Sigma laboratories centrifuge 3K30). Enzyme (10 µL) was added to each plate well (containing 40 µL of buffers) for the assays chymotrypsin-, trypsin-, and elastase-like proteinases. Stock substrates of BAρNA (100 mg/mL in DMSO), SAAPFρNA (100 mg/mL in DMF), and SAAPLρNA (100 mg/mL in DMF) were diluted to 1.0 mg/mL with an assay buffer. The total volume of the substrate was 50 µL. The reaction mixture was immediately incubated at 37 °C for 15 min and then stopped with 50 µL 30% acetic acid. Nitroaniline was measured at 405 nm using an ELISA plate reader. An assay mixture with a denaturation enzyme instead of a live enzyme was used as the blank well. Specific proteinase activities were expressed in OD/min mg protein in triplicate (for three substrates).

### 2.11. Determination Effects of T. vulgaris and O. basilicum Extract from Cell Suspension and Pure Compounds on Metalloproteinase Specific Activity Assays

Metalloproteinase activity using azocasein as the substrate was assessed in 4th midgut instars larvae homogenate of *R. ferrugineus*. The 4th midgut larvae (10 larvae) were pulled out gently, excised, washed with saline solution (0.9% NaCl) repeatedly to remove foodstuff and then homogenized in a 500 µL assay buffer. The midgut instar was homogenized in a 500 µL protease assay buffer [50 mM HEPS (*N*-2-hydroxyelthyl piperazin-*N*′-2-ehtanesulphonic acid), pH 8.0, 5 mM dithiothreitol (DTT), and 0.1% Triton X-100]. The homogenates were centrifuged at 5000× *g* for 30 min using the Sigma 3k30 cooling centrifuge. The supernatants were used for estimation of enzyme activity and protein concentration. Ten microliters of the supernatant per assay were incubated in a total volume of 60 µL of assay buffer (pH 8) for 20 min at 37 °C before the addition of 200 µL of azocasein (2%, *w*/*v* in assay buffer). In all cases, enzyme samples of 10 µL and extracts from cell suspension after 40 days (1, 10, 50, 100, 500, 1000, and 5000 mg/L) were pre-incubated for 10 min (10 min for EDTA) before the substrate was added. 

The reaction was stopped with 300 µL of cold 10% trichloroacetic acid (TCA) and proceeded for 180 min at 37 °C. The reaction mixture was centrifuged at 5000× *g* for 20 min using the Sigma 3k30 cooling centrifuge, 10 µL of 10 N NaOH was added to the supernatant, and absorbance at 450 nm was measured using an ELISA plate reader. An assay mixture without an enzyme was used as the blank. The specific activity was calculated as OD450 mg^−1^·protein^−1^·h^−1^ (OD mg^−1^·protein·min^−1^) and a blank sample without an enzyme solution.

### 2.12. Determination Effects of T. vulgaris and O. basilicum Extract from Cell Suspension and Pure Compounds on Cysteine Proteinase Specific Activity Assays

Cysteine proteinase activity was assessed using Z-Phe-Arg-MNA as a substrate in 4th midgut instar larval homogenate of *R. ferrugineus*. The 4th midgut larvae (10 larvae) were pulled out gently, excised, washed with saline solution (0.9% NaCl) repeatedly to remove foodstuff, and then homogenized in a 500 µL assay buffer. The midgut instar was homogenized in a 500 µL protease assay buffer [50 mM HEPS (*N*-2-Hydroxyelthyl piperazin-*N*′-2-ehtanesulphonic acid), pH 8.0, 5 mM DTT, and 0.1% Triton X-100]. The homogenates were centrifuged at 5000× *g* for 30 min using the Sigma 3k30 cooling centrifuge. The supernatants were used for the estimation of the total proteolytic enzyme activity and the protein concentration. Ten microliters of supernatant per assay were incubated in a total volume of 60 µL of assay buffer (pH 8) for 30 min at 37 °C before the addition of 100 µL of a 0.5 mM substrate. In all cases, enzyme samples of 10 µL and extracts from cell suspension after 40 days (50, 100, 500, 1000, and 5000 mg/L) were pre-incubated (10 min) with iodoacetic acid before the addition of the substrate. The reaction was stopped after incubation (for 60 min at 37 °C) by adding 1.5 mL of 5 mM mersalyl, 2% Tween 20, and 0.02 mg/mL fast garnet reactive solution and then centrifuged (5000× *g*, 5 min), and the absorbance was measured at 520 nm. The proteolysis was defined as the OD520/60 min/mg of midgut protein using an ELISA plate reader. An assay mixture without an enzyme was used as the blank. 

Specific protease inhibitors were added to the reactions of proteolytic activity determination assays, and the inhibitors were: PMSF, a general serine proteinase inhibitor; TLCK, a trypsin-like serine proteinase inhibitor; TPCK, a chymotrypsin-like serine proteinase inhibitor; an iodoacetic acid-like cysteine proteinase inhibitor; an EDTA-like metalloproteinase inhibitor; and leupeptin, a general proteinase inhibitor. The 4th midgut larval homogenates of a lab strain were prepared as described above in the assay for enzyme activity determination. The inhibition assay was carried out on a microplate assay as described above. A series of inhibitor concentrations was prepared in order to establish the maximum inhibition by each inhibitor. The leupeptin (0.01, 0.05, 0.1, and 1.0 mM), PMSF (0.1, 1.0, 10, and 50 mM), TLCK and TPCK (0.01, 0.05, 0.1, 1, 50, and 100 mM), EDTA (0.1, 1, 10, 50, and 100 mM), and iodoacetic acid concentrations (0.01, 0.05, 0.1, and 1 mM) were dissolved in the assay buffers. 

To identify the proteases present in the *R. ferrugineus* 4th midgut larval instar homogenate preparations, different mechanistic class inhibitors were tested. In all protease types, enzyme samples of 10 µL and inhibitors or extracts from the cell suspension after 40 days or pure compounds (thymol, *p*-cymene, γ-terpinene, β-caryophyllene, linalool, estragole, β-terpineol, ocimene, 1,8-cineole, germacrene D, and eugenol) (1, 10, 50, 100, 500, 1000, and 5000 mg/L) were pre-incubated for 10 min before adding the substrate. An assay mixture without inhibitors was used as control. Absorbance was measured using an ELISA plate reader at different nm according to the type of enzymes. Percent activity of the control for each inhibitor was calculated to each enzyme. 

### 2.13. Statistical Design

The statistical analysis and a probit analysis according to Finney [43] was performed using SPSS 25.0 software (Statistical Package for Social Sciences, Boston, MA, USA). All quantitative estimations of the tissue culture parameters were based on 3 replicates of callus and cell suspension, and the values were expressed as mean ± SD. All the quantitative estimations of toxicity parameters were based on 10 replicates, and the values were expressed as mean ± standard error. A regression was made of the mortality against the dose, and the obtained median dose was converted to an LC_50_ value (µg/mL). The 95% confidence limits for the range of LC_50_ were determined by least-square regression analysis of the relative growth rate (% control) against the logarithm of the extract concentration. The data of each growth weight and enzyme activity were subjected to a one-way analysis of variance (ANOVA). Mean separations were performed by the Student–Newman–Keuls (SNK) test, and differences at *p* ≤ 0.05 were considered significant.

## 3. Results

### 3.1. Cell Suspension and Callus Initiation and Maintenance

**Figure 1:**
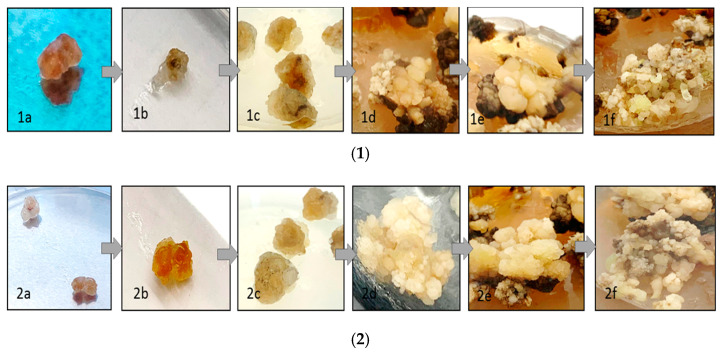
Callus tissues and callus induction in the MS medium containing (kinetin (0.5 mg/L), 2,4-D (0.1 mg/L), NAA (0.1 mg/L), and IBA (1 mg/L) as plant growth regulators (PGRs) and 3% (*w*/*v*) sucrose; (**1**) callus induction of *T. vulgaris* (interval 7 days) and (**2**) callus induction of *O. basilicum* in different growth stages (interval 7 days).

Cell suspension culture was started with embryogenic calli and then transferred at the age of 5–6 weeks to the cell suspension media. It is noticeable that the LS liquid media appeared steadily and produced more somatic embryos than what was present in the solid media used for that purpose. The maximum weights of the cell suspension were obtained after 40 days (6.24 and 5.03 g/200 mL media without infection and 7.24 and 5.35 g/200 mL media with infection of *O. basilicum* and *T. vulgaris*, respectively). It was also noted that MS and calli have an oxidative process, where a rapid brown color appears, and when using macronutrients in full quantities, only a few of the formed physical somatic embryos developed (Figure 1). 

**Figure 2:**
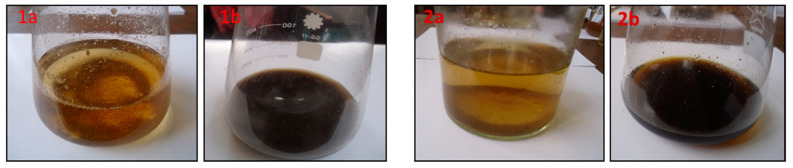
Cell suspension from callus through somatic embryogenesis using LS after 7 days (**1a**,**2a**) and the end of induction (40 days) (**1b**,**2b**) in (**1**) *T. vulgaris* and (**2**) *O. basilicum.*

At the beginning of initiation, the liquid media and calli were characterized by a brown color, and the color then became darker, indicating that the formation of phenolic constituents in the media and the initiation process are time-dependent (Figure 2). As shown in Table S1, infection by *V. dahlia*e significantly increased the weight of the cell suspension of *T. vulgaris* and *O. basilicum*, and the weights of the callus were higher than those obtained from the cell suspension in all different ages of callus. The maximum weights of callus after 40 days were 7.11 and 8.25 in media without infection of *T. vulgaris* and *O. basilicum,* respectively, and the weight values increased with the age of the callus and infection by *V. dahliae*. The infected calli of *T. vulgaris* and *O. basilicum* were 7.80 and 10.51 g, respectively (Table S1).

### 3.2. Chemical Content Analyses and Chemical Composition from Volatile Extract in O. basilicum and T. vulgaris

Total volatile phenolic content (mg/g DW) in *T. vulgaris* and *O. basilicum* increased especially during inoculation with *V. dahliae* in the callus until the end of the test period (40 days) (Table 1). The values obtained in Table 1 show the total volatile phenolic content in the *O. basilicum* in the callus, and the cell suspension was greater than that of *T. vulgaris*. The total volatile phenolic content in the infected callus and cell suspension of *O. basilicum* was 11.02 and 25.74 mg/g dried weight, respectively. The previous values were less than that in the absence of a callus and cell suspension of *O. basilicum* without infection by *V. dahliae* (7.12 and 14.24 mg/g dried weight, respectively). The *T. vulgaris* total volatile phenolic content shown in Table 1 shows that values are lower than those obtained from *O. basilicum* with and without infection by *V. dahliae* in the callus and cell suspension. The mg of the total volatile phenolic/g dried weight of non-infected callus and cell suspension were 4.02 and 10.04 for *O. basilicum*, respectively. Those previous values of total volatile phenolic content from *T. vulgaris* increased with infection by *V. dahlia*e to 6.84 and 15.36 mg/g DW of callus and cell suspension, respectively. Overall, the total volatile phenolic content showed a significant difference with and without infection by *V. dahliae* in *T. vulgaris* and *O. basilicum* (Table 1).

As shown in Table 2 and Figure S1, the percentage of oxygenated monoterpenes was the highest value obtained in the volatile extracts of *T. vulgaris and O. basilicum* (52.45% and 49.89%, respectively). Accordingly, the family percentages of volatile extracts of *T. vulgaris* in descending order are the oxygenated monoterpenes (52.45%), monoterpene hydrocarbons (30.5%), sesquiterpene hydrocarbons (8.72%), oxygenated sesquiterpenes (1.8%), and phenylpropanoids (0.3%).

The chemical composition of the *O. basilicum* volatile extract showed differences from the previous data obtained in *T. vulgaris*: the values were 49.89%, 19.92%, 17.63%, 4.2%, and 1.9% for oxygenated monoterpenes, sesquiterpenes hydrocarbons, monoterpenes hydrocarbons, phenylpropanoids, and oxygenated sesquiterpenes, respectively.

Table 2 shows that thymol was the most abundant compound in the oxygenated monoterpenes of the *T. vulgaris* volatile extract with 40.5%, followed by linalool at 4%, carvacrol methyl ether at 1.7%, thymol methyl ether at 1.7%, and geraniol acetate at 1%. All compounds appeared only in *T. vulgaris* and not in *O. basilicum*, except linalool with a value of 1.2%. On the other hand, oxygenated monoterpenes of the *T. vulgaris* volatile extract showed 18 compounds, 12 compounds of which showed values of less than 1%. 

The monoterpene hydrocarbon family was the second highest group of volatile extracts of *T. vulgaris* with a value of 30.5% and containing 12 compounds (Table 2). *p*-cymene (17.3%) was the major compound of this family that appeared only in volatile extracts of *T. vulgaris*, followed by γ-terpinene at 9.1%, while the remaining compounds in monoterpene hydrocarbons did not exceed 0.5% of the total volatile extracts.

The *T. vulgaris* extract contained sesquiterpene hydrocarbons (8.72%) of total volatile extracts. β-caryophyllene appeared at 6.28%, representing 72% of all sesquiterpene hydrocarbons. Oxygenated sesquiterpenes of the *T. vulgaris* volatile extract appeared in 1.8% of the total compounds with four compounds (Table 2). Phenylpropanoids appeared in a value not more than 0.3%, represented only by chavicol, and the value of non-terpene derivatives did not exceed 1.62% (Table 2).

Oxygenated monoterpenes were found in the largest proportion of the *O. basilicum* volatile extract (49.89%) and consisted of 15 compounds (Table 2). Estragole appeared at 22.38% as a major component of oxygenated monoterpenes in only the *O. basilicum* volatile extract, followed by β-terpineol, 1,8-cineole (eucalyptol), nerol, and linalool at 12.37%, 7.24%, 1.6%, and 1.2%, respectively. Of the sesquiterpene hydrocarbons of the *O. basilicum* volatile extract, α-guaiene appeared as a major compound in this family with a value of 4.8% of the total *O. basilicum* volatile extract (Table 2). Sesquiterpene hydrocarbons also contained germacrene D, β-bergamotene, β-farnesene, and α-humulene in proportions of 4.2%, 2.3%, 2.3%, and 1.52%, respectively. Monoterpene hydrocarbons appeared as the third largest group in the volatile extract composition of *O. basilicum* with a value of 17.63%. They were composed of 13 compounds (Table 2), and the most significant was (*E*)-β-ocimeme with a value of 11.96%. Eugenol, methyl eugenol, and chavicol appeared as phenylpropanoid compounds in the *O. basilicum* volatile extract with values of 3.7%, 0.4%, and 0.1%, respectively. Oxygenated sesquiterpenes α-eudesmol and τ-cadinol were found in amounts of 1.8% and 0.1%, respectively, of the total volatile extract (Table 2). Finally, the value of residual compounds of the non-terpene derivative class in the *O. basilicum* volatile extract did not exceed 0.7% (Table 2).

**Figure 3:**
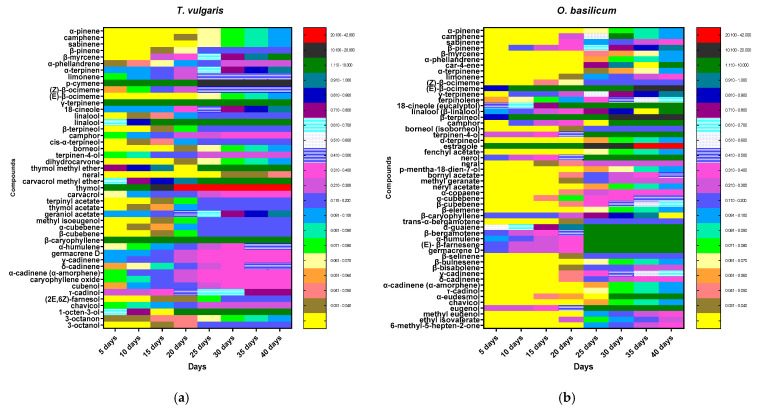
Heatmap of (**a**) *T. vulgaris* and (**b**) *O. basilicum* cell suspension extracts during initiation time (40 days) analyzed by GC-MS. Secondary metabolites were displayed as colors ranging from yellow to red (as shown in the key).

Data presented in Figure 3 show that the thymol (40.5%), *p*-cymene (17.3%), γ-terpinene (9.1%), β-caryophyllene (6.12%), and linalool (4.0%) of *T. vulgaris* and *O. basilicum* estragole (22.38%), (*E*)-β-ocimene (12.69%), β-terpineol (12.37%), 1,8-cineole (eucalyptol) (7.24%), germacrene D (4.2%), and eugenol (3.7%) of *O. basilicum* increased slowly in the first 25, days rapidly in cell suspension and continued to increase quickly in the last 15 days until the end of the test period (40 days) (Figure 3).

### 3.3. Insecticidal and Antifeedant Activity of Extract against Red Palm Weevil (R. ferrugineus) Adults and Larvae

Data presented in Table 3 show the effectiveness of volatile extracts that were used in the current study on adult red palm weevils (*R. ferrugineus)*. The LC_50_ values were 1032 and 1246 µg/mL for *T. vulgaris* and *O. basilicum*, respectively. Probit analysis estimated that the most active volatile extract against *R. ferrugineus* adults was *T. vulgaris* with an LC_50_ of 1032 µg/mL, 95% confidence limits of 891–1223, followed by *O. basilicum* with an LC_50_ of 1246 µg/mL, confidence limits 1046–1501. Topical application showed that LD_50_ values (µg/larva) were 11.4 and 14.6 of *T. vulgaris* and *O. basilicum* volatile extracts, respectively.

### 3.4. Effects of T. vulgaris and O. basilicum Extract from Cell Suspension on Serine, Cysteine, and Metalloproteinase Specific Activity Assays 

**Figure 4:**
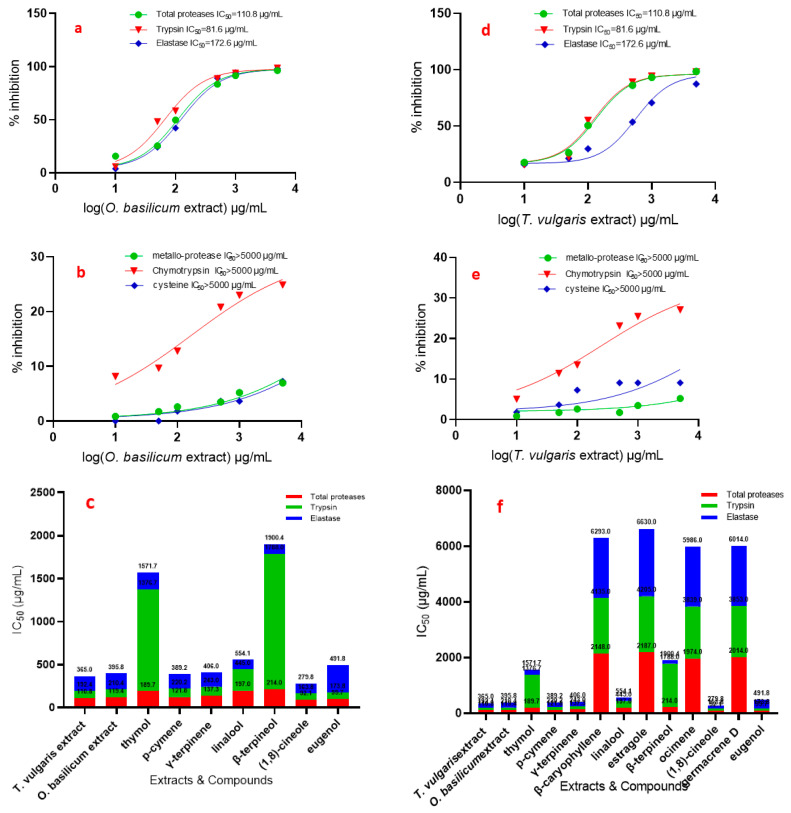
IC_50_ (in vitro) of *T. vulgaris* and *O. basilicum* extract from cell suspensions and compounds against total proteases, trypsin, chymotrypsin, elastase, cysteine, and metallo-protease of *R. ferrugineus* midgut. (**a**) IC_50_ of *O. basilicum* against total proteases, trypsin, elastase and (**b**) IC_50_ of *O. basilicum* against metallo-protease, chymotrypsin and cysteine, (**c**,**f**) IC_50_ of *T. vulgaris* and *O. basilicum* extract, thymol, *p*-cymene, γ-terpinene, linalool, β-terpineol, 1,8-cineole and eugenol against total proteases, trypsin, elastase, (**d**) IC_50_ of *T. vulgaris* against total proteases, trypsin, elastase, (**e**) IC_50_ of *T. vulgaris* against metallo-protease, chymotrypsin and cysteine.

Protease activity towards azocasein in the 4th instar midgut homogenate preparation in larval instar is represented in Figure 4. The specific activity was expressed as OD/mg protein min for larval midgut preparation. It is evident from the activity data, that the specific activity of 4th instar midgut preparation showed the highest activity.

Data presented in Figure 4 show the effect of the used serial concentrations of *T. vulgaris* and *O. basilicum* volatile extracts on total protease activity from the midgut of the 4th instar larvae of *R. ferrugineus*. The data showed that the effect of serial doses (10, 50, 100, 500, 1000, and 5000 µg/mL) of *T. vulgaris* and *O. basilicum* volatile extract on IC_50_ values was significant compared to untreated proteases from larvae midgut. Data (Figure 4) clearly indicated that the IC_50_ of the 4th instar larvae midgut increased gradually with increasing concentrations. IC_50_ values were 110.8 and 119.4 µg/mL for *T. vulgaris* and *O. basilicum* volatile extract, respectively. The results showed that the most active volatile extract was *T. vulgaris* with an IC_50_ value of 110.8 µg/mL, followed by *O. basilicum* extract with an IC_50_ value of 119.4 µg/mL. Data presented in Figure 4 show that purified proteases from midgut 4th larval instars were strongly inhibited by the total protease inhibitor PMSF. 

Relative trypsin, chymotrypsin, and elastase-like proteinase activity and inhibition by extracts in 4th instar midgut homogenate preparations are presented in Figure 4.

The specific activities of the serine proteinases in total homogenate preparations are similar. The effect of all doses on the IC_50_ rate was significant with all tested *T. vulgaris* and *O. basilicum* extracts, compared to untreated proteases from larvae midgut. The IC_50_ of 4th instar larvae midgut of *R. ferrugineu* increased gradually with increasing concentrations of the extracts: doses of 10, 50, 100, 500, 1000, and 5000 µg/mL. 

Chymotrypsin-like serine proteinases show a different trend from that of the serine proteinase specific activity; the IC_50_ values of trypsin and chymotrypsin are presented in Figure 4, which shows that all extracts have the least inhibition in trypsin and chymotrypsin where IC_50_ > 5000 µg/mL.

Trypsin-like serine proteinases have a different trend from that of the chymotrypsin-like serine proteinase effect by the *T. vulgaris* and *O. basilicum* extracts; the IC_50_ values of *T. vulgaris* and *O. basilicum* extract are presented in Figure 4a,b,d,e, which shows that the inhibition variation in midgut homogenate preparations significantly differ. Trypsin-like serine proteinase activity in OD/mg protein min from 4th instar midgut preparation was 4.10. The most active extract was *T. vulgaris* with an IC_50_ of 81.6 µg/mL. *O. basilicum* extract was less active, with an IC_50_ of 91 µg/mL. Inhibition by these extracts is clearly shown in trypsin-like serine proteinases from 4th midgut preparation. Cysteine and metalloprotease have a different trend from that of the elastase-like serine proteinase inhibition of *T. vulgaris* and *O. basilicum* extracts; the IC_50_ values are presented in Figure 4, which shows that all extracts have the least inhibition where IC_50_ > 5000 µg/mL.

Figure 4c,f shows the effect of pure compounds on total protease activity (in vitro) from the midgut of 4th instar larvae of *R. ferrugineus*. The effect of different doses on the IC_50_ rate was significant with all tested compounds, compared to the untreated larvae. 1,8-Cineole, eugenol, *p*-cymene, γ-terpinene, thymol, linalool, and β-terpineol are most active against total proteases, with IC_50_ values of 92.1, 99.7, 121.8, 137.3, 189.7, 197, and 214 µg/mL, respectively. 1,8-Cineole, eugenol, *p*-cymene, and γ-terpinene have a more specific effect against trypsin, with IC_50_ values of 71.7, 74.1, 98.4, and 105.7µg/mL, respectively.

The elastase activity shows the inhibition roles of linalool, β-terpineol, 1,8-cineole, γ-terpinene, *p*-cymene, and thymol with IC_50_ values of 109.1, 112.4, 116, 163, 169, and 195 µg/mL, respectively.

To evaluate the role of different gut proteases in 4th larval instars by protease inhibitors, they were included in the proteolysis assays. Table 4 shows that purified proteases from *R. ferrugineus* midgut 4th larval instars were strongly inhibited by the total proteases, serine proteinases, and elastase inhibitors. Moreover, the trypsin-like serine proteinase inhibitor TLCK, the chymotrypsin-like serine proteinase inhibitor TPCK, and iodoacetic acid as a cysteine protease inhibitor significantly inhibited the midgut instar. 

## 4. Discussion

In the present research, we investigated the metabolomics of total volatile extracts from cell suspensions of *O. basilicum* and *T. vulgaris*. It can be concluded that 1,8-cineole, eugenol, *p*-cymene, γ-terpinene, thymol, and linalool have high insecticidal activity against *R. ferrugineus*. The results showed that the most active volatile extracts against total proteases, trypsin-like serine proteinases, and elastases were *T. vulgaris*, followed by *O. basilicum* extracts, using both in vitro assays. The production of volatile secondary metabolites including phenolic compounds increased rapidly in cell suspensions of *T. vulgaris* and *O. basilicum,* especially during inoculation with *V. dahlia*e.

It was found that the *T. vulgaris* extract is the most active and effective, not only by increasing deterrence on the larvae of *R. ferrugineus*, but also by inhibiting feeding. In addition, the *O. basilicum* extract also exhibited a lower biological activity and efficiency compared to those of *T. vulgaris*. However, their effective antifeedant activity on the adult is the most pronounced and interesting. Moreover, as we demonstrated in our results, the highest values of antifeedant activity were achieved in choice conditions, and the sensitivity of the no-choice test was higher than in the choice test. 

The *Thymus vulgaris* extract showed significant insecticidal activity, which could be attributed to the diversity of bioactive metabolite content. The major compounds thymol, *p*-cymene, γ-terpinene, β-caryophyllene, and linalool of *T. vulgaris* and estragole, (*E*)-β-ocimene, β-terpineol, 1,8-cineole (eucalyptol), and germacrene D of *O. basilicum* increased slowly in the first 25 days, rapidly in cell suspension and continued to increase quickly in the last 15 days until the end of the test period (40 days). 

In addition, the results demonstrated that the activity was elevated, with an increase in previous compounds and a total increase in monoterpene hydrocarbons content of 30.5% more than that found in *O. basilicum* extract (17.63%). Data show the effect of pure compounds on total protease activity (in vitro). The effect of different doses on the IC_50_ rate was significant with all tested compounds.

The effect of pure compounds on total protease activity (in vitro) from the midgut of 4th instar larvae of *R. ferrugineus* explains the activity of *T. vulgaris* and *O. basilicum*. *Thymus vulgaris* extract activity against elastase entirely depends on the content of linalool and thymol (4% and 40.5%) with IC_50_ values of 109.1 and 195 µg/mL, and that against trypsin and elastase depends on 1,8-cineole, *p*-cymene, and γ-terpinene (1%, 17.3%, and 9.1%) with IC_50_ values of 71.7, 98.4, and 105.7 µg/mL for trypsin and 116, 169, and 163 µg/mL for elastase, respectively. 

However, *O. basilicum* extract activity against elastase is related to β-terpineol and linalool (12.37% and 1.2%) with IC_50_ values of 1121.4 and 109.1 µg/mL, respectively. On the other hand, eugenol (3.7%) showed activity against trypsin only with an IC_50_ value of 74.1 µg/mL. Trypsin and elastase were inhibited with a 1,8-cineole content of 7.24% and IC_50_ values of 71.7 and 116 µg/mL and with a γ-terpinene content of 1% with IC_50_ values of 105.7 and 163 µg/mL, respectively.

1,8-Cineole, eugenol, *p*-cymene, and γ-terpinene showed a specific inhibition effect against trypsin with IC_50_ values of 71.7, 74.1, 98.4, and 105.7 µg/mL, respectively. The elastase activity showed high inhibition roles in linalool, β-terpineol, and 1,8-cineole, with IC_50_ values of 109.1, 112.4, and 116 µg/mL, respectively, and moderate inhibition roles in γ-terpinene, *p*-cymene, and thymol (IC_50_ values of 163, 169, 195 µg/mL, respectively). As a result, the activities of the extracts are related to the inhibition role of compounds (Figure 4c,f). 

It can be concluded that thymol, *p*-cymene, γ-terpinene, β-caryophyllene, and linalool have high insecticidal activity against *R. ferrugineus*. However, the targets and mechanism of action in insects for these extracts remain unknown as antifeedant compounds. As described in the literature, mechanisms could include the disruption of feeding physiology, repellency, or chronic toxicity possibly related to the insecticidal action [44,45].

The most prominent secondary metabolites were monoterpenes, sesquiterpenes, and phenylpropanoid derivatives in the volatile extracts of both *T. vulgaris* and *O. basilicum*. The phenolic compounds were the main step in the biosynthesis process for phenylpropanoids, monoterpenes, sesquiterpenes, and lignin precursors [46,47]. It is noted that these secondary compounds are considered as major components after the deposition on cells as an important defense against pathogen infection [48,49]. There is a significant group of high phenolic compounds and PGR-like indole acetic acids (IAA) which can manipulate catabolism demolition [50], so PGR is important for the regulation of plant growth, cell characterization, and plant cell differentiation [51].

Hence, new methods such as the green biosynthesis of biologically active secondary metabolites are currently in high demand [52,53,54]. Moreover, we find that many previous records indicate that the use of cell suspension culture, compared with the callus, is a more effective and rapid method for increasing bioactive compound production, as it includes rapid response, cell division, and ease of application [53,54].

Total proteolytic activity from the larval instars of the *R. ferrugineus* in the gut juice was determined using a protein substrate (azocasein), and the activity was estimated by performing the reactions using a slightly alkaline mixture (pH 8.0), as it is physiologically related to the pH of the insect midgut, and a DTT is used as an activator [55]. 

The phenomena emerging from the differences in the *T. vulgaris* and *O. basilicum* extract’s volatile secondary metabolites towards the inhibition of proteases in vitro assay (Table 4) clarified the importance of proteinases in the mode of action [56]. The results suggest that toxicity can be related with thymol, *p*-cymene, γ-terpinene, and linalool in the *T. vulgaris* extract and may confirm the importance of proteinases, because this potentiation is due to the activation of proteinases more so than estragole (22.38%), (*E*)-β-ocimene (12.69%), or germacrene D (4.2%) in the *O. basilicum* extract’s volatile secondary metabolites.

It was also clear that the maximum callus weight obtained after 40 days of germination, and the mean weight of callus gradually increased as the age increased. All volatile phenolic compounds increased rapidly in all *T. vulgaris* and *O. basilicum* specimens, especially during the inoculation with *V. dahliae* in the callus, and continued to increase until the end of the test period (40 days). The concentrations of the total volatile phenolic constituents in *T. vulgaris* and *O. basilicum* were assessed, and the main compounds that were found were oxygenated monoterpenoids (52.45% and 49.89%, respectively), specifically thymol, *p*-cymene, γ-terpinene, β-caryophyllene, and linalool in *T. vulgaris* volatile extract and estragole, β-terpineol, (*E*)-β-ocimene, 1,8-cineole (eucalyptol), germacrene D, and eugenol in *O. basilicum* volatile extract. 

Finally, the most active extract against the red palm weevil (*R. ferrugineus*) was *T. vulgaris* according to the LC_50_ values, followed by *O. basilicum*. The antifeedant activity for the *T. vulgaris* and *O. basilicum* volatile extract was clearly effective. Inhibition by *T. vulgaris* and *O. basilicum* was clearly shown in the proteinases obtained from the 4th midgut preparation. These results provide a groundwork for new ways that these compounds can be used for the composition of biochemical markers that determine how resistant various plants are to pest infestation.

## 5. Conclusions

In summary, the findings demonstrate that volatile secondary metabolites from *T. vulgaris* and *O. basilicum* extract could be used as bio-insecticides against the red palm weevil. The results showed a relationship between the volatile secondary metabolites and their use, which could be attributed more to the phenolic compounds in *T. vulgaris* than in *O. basilicum*. The study is of great need for future field applications for further understanding, and for evaluating the effectiveness and benefits derived from the use of such volatile secondary metabolites. However, the production of these secondary metabolites can be implemented on a large scale using an easy and clean technique (cell suspension).

## Figures and Tables

**Table 1:** Chemical content analyses from volatile extract in *O. basilicum* and *T. vulgaris* from callus and cell suspension with and without infection by *V. dahliae.*

Callus and Cell Suspension	TVPC (mg of Gallic Acid/g DW)	
Species	*O. basilicum*	*T. vulgaris*
Callus without infection	7.12 ± 0.11	4.02 ± 0.13
Callus with infection	11.02 ± 0.15	6.84 ± 0.11
Cell suspension without infection	14.24 ± 0.12	10.04 ± 0.22
Cell suspension with infection	25.74 ± 0.20	15.36 ± 0.24

Mean ± Standard deviation (SD), *n* = 3, total volatile phenolic content (TVPC).

**Table 2:** Chemical composition of monoterpene hydrocarbons, oxygenated monoterpenes, sesquiterpene hydrocarbons, oxygenated sesquiterpenes, total phenylpropanoids, classes, and the total identified extract from the cell suspensions of *T. vulgaris* and *O. basilicum.*

No.	Compounds	RI (exp)	RI (lit)	*T. Vulgaris*	*O. Basilicum*
				Relative Abundance %	
**Monoterpene Hydrocarbons**					
1	α-pinene	937–934 *	932	0.1 ± 0.02	0.1 ± 0.02
2	camphene	952–948 *	946	0.1 ± 0.02	0.1± 0.02
3	sabinene	973–976 *	973	0.1 ± 0.02	0.4 ± 0.06
4	β-pinene	977–978 *	977	0.2 ± 0.02	1 ± 0.02
5	β-myrcene	991–992 *	988	1.2 ± 0.2	0.1 ± 0.04
6	α-phellandrene	1005	1002	0.3 ± 0.02	0.1 ± 0.04
7	car-4-ene	1009	1004	-	1.2 ± 0.1
8	α-terpinene	1017–1018 *	1014	1 ± 0.2	0.1 ± 0.1
9	limonene	1030	1224	0.5 ± 0.06	0.4 ± 0.1
10	*p*-cymene	1026–1023 *	1023	17.3 ± 0.4	-
11	(Z)-β-ocimeme	1038–1040 *	1032	0.5 ± 0.06	0.2 ± 0.02
12	(*E*)-β-ocimeme	1049–1050 *	1044	0.1 ± 0.03	11.96 ± 0.2
13	γ-terpinene	1060–1061 *	1067	9.1 ± 0.5	1.0 ± 0.2
14	terpinolene	1088	1086	-	0.7 ± 0.1
**Total Monoterpene Hydrocarbons Identified %**				**30.5 ± 1.55**	**17.63± 1.02**
**Oxygenated Monoterpenes**					
1	1,8-cineole (eucalyptol)	1031–1026 *	1031	1 ± 0.1	7.24 ± 0.4
2	linalool oxide	1094	1094	0.2 ± 0.04	-
3	linalool (β-linalool)	1099–1100 *	1095	4.0 ± 0.8	1.2 ± 0.2
4	β-terpineol	1130	1130	0.2 ± 0.05	12.37 ±0.87
5	camphor	1145–1150 *	1141	0.4 ± 0.03	1.4 ± 0.2
6	*cis*-α-terpineol	1143	1143	0.2 ± 0.04	-
7	borneol (isoborneol)	1167–1141 *	1165	0.1 ± 0.02	0.2 ± 0.05
9	terpinen-4-ol	1177–1182 *	1174	0.3 ± 0.02	2 ± 0.3
10	dihydrocarvone	1179	1179	0.1 ± 0.02	-
11	α-terpineol	1189	1186	-	0.1 ± 0.04
12	estragole	1199	1199	-	22.38 ± 0.7
13	fenchyl acetate	1214	1214	-	0.1 ± 0.02
14	nerol	1228	1227	-	1.6± 0.2
15	thymol methyl ether	1235–1161 *	1235	1.7 ± 0.2	-
16	neral	1244	1244	0.05 ± 0.02	0.3 ± 0.1
17	carvacrol methyl ether	1248–1165 *	1245	1.7 ± 0.1	-
18	*p*-mentha-1,8-dien-7-ol	1261	1261	-	0.1 ± 0.04
19	thymol	1264–1265 *	1266	40.5± 0.86	-
20	bornyl acetate	1285	1284	-	0.5 ± 0.02
21	carvacrol	1299–1293 *	1298	0.4 ± 0.04	-
22	methyl geranate	1321	1319	-	0.3 ± 0.03
23	terpinyl acetate	1333	1333	0.2 ± 0.03	-
24	thymol acetate	1349	1352	0.2 ± 0.04	-
25	neryl acetate	1364	1359	-	0.1 ± 0.04
26	geraniol acetate	1370	1368	1 ± 0.2	-
27	methyl isoeugenol	1492	1495	0.2 ± 0.02	-
**Total Oxygenated Monoterpenes Identified %**				**52.45± 2.63**	**49.89± 2.88**
**Sesquiterpene Hydrocarbons**					
1	α-copaene	1376	1374	-	0.4 ± 0.03
2	α-cubebene	1385–1386 *	1387	0.2 ± 0.04	0.5 ± 0.02
3	β-cubebene	1389–1494 *	1387	0.2 ± 0.03	0.7 ± 0.05
4	β-elemene	1391	1389	-	0.1 ± 0.03
5	β-caryophyllene	1424–1433 *	1424	6.12 ± 0.1	1.1 ± 0.1
6	*trans*-α-bergamotene	1435	1432	-	0.2 ± 0.03
7	α-guaiene	1439	1437	-	4.8 ± 0.2
8	β-bergamotene	1441	1438	-	2.3 ± 0.2
9	α-humulene	1455	1452	0.5 ± 0.05	1.52 ± 0.04
10	(*E*)-β-farnesene	1457	1454	-	2.3 ± 0.08
11	germacrene D	1481	1484	0.4 ± 0.02	4.2 ± 0.2
12	β-selinene	1486	1489	-	0.2 ± 0.03
13	β-bulnesene	1505	1508	-	0.1 ± 0.02
14	β-bisabolene	1509	1512	-	0.3 ± 0.06
15	γ-cadinene	1513–1527 *	1513	0.4 ± 0.03	0.7 ± 0.02
16	δ-cadinene	1525–1535 *	1522	0.5 ± 0.05	0.4 ± 0.04
17	α-cadinene (α-amorphene)	1538–1487 *	1537	0.4 ± 0.02	0.1 ± 0.02
**Total Sesquiterpene Hydrocarbons (SH) Identified %**				**8.72 ± 0.34**	**19.92 ± 1.17**
**Oxygenated Sesquiterpenes**					
1	caryophyllene oxide	1509–1512 *	1507	0.4 ± 0.04	-
2	cubenol	1515	1514	0.4 ± 0.03	-
3	τ-cadinol	1640	1638	0.8 ± 0.05	0.1 ± 0.02
4	α-eudesmol	1653	1652	-	1.8 ± 0.1
5	(*2E,6Z*)-farnesol	1715	1712	0.2 ± 0.02	-
**Total Oxygenated Sesquiterpenes (OS) Identified (%)**				**1.8 ± 0.14**	**1.9± 0.12**
**Phenylpropanoids**					
1	chavicol	1256–1250 *	1247	0.3 ± 0.05	0.1 ± 0.03
2	eugenol	1357	1356	-	3.7 ± 0.3
3	methyl eugenol	1406	1402	-	0.4 ± 0.02
**Total Phenylpropanoids (PP) Identified (%)**				**0.3 ± 0.05**	**4.2 ± 0.35**
**Non-Terpene Derivatives**					
1	ethyl *iso*valerate	853	856	-	0.3 ± 0.02
2	1-octen-3-ol	981	981	1.32 ± 0.1	-
3	6-methyl-5-hepten-2-one	985	988	-	0.4 ± 0.02
4	3-octanon	989	988	0.1 ± 0.01	-
5	3-octanol	996	996	0.2 ± 0.03	-
**Total Non-Terpene Derivatives (NT) Identified (%)**				1.62 ± 0.14	0.7 ± 0.04
Total Identified (%)				95.39	94.24

Value was obtained from 3 replicates (Mean ± Standard deviation (SD), *n* = 3); RI (exp): relative retention index determined on VF-5ms fused silica capillary column; * RI (exp) of *T. vulgaris* if the value is different from that obtained from the *O. basilicum*; RI (lit) relative retention index from MS libraries (Wiley); National Institute of Standards and Technology (NIST); Separation profiles of extracts after 40 days were explaining in Supplementary Figure S1.

**Table 3:** Probit analysis of mortality for a laboratory-susceptible population of *R. ferrugineus* adults and 4th larvae after exposure to *T. vulgaris* and *O. basilicum* extract.

Extract	Adult				4th Larvae			
	LC_50_ (µg/mL) 95% CF	Slope	Chi Square	*p*	LD_50_ (µg/Larvae) 95% CF	Slope	Chi Square	*p*
*T. vulgaris*	1032 (891–1223)	25.4 ± 1.41	51.8	<0.01	11.4 (9.97–12.74)	9.32 ± 0.64	47.21	<0.01
*O. basilicum*	1246 (1046–1501)	2.51 ± 0.21	43.7	<0.01	14.6 (12.32–15.94)	1.24 ± 0.23	41.58	<0.01

LC_50_: lethal concentration; LD_50_: lethal dose; CF: Confidence Limits; Data are expressed (±SE) based on five replicates per tested concentration; *n* = 10.

**Table 4:** In vitro specific activity of protease enzymes (OD/mg protein min) in *R. ferrugineus* larval instars ± SE treated with specific inhibitors.

Inhibitors	Conc. (mM)	In Vitro (OD/mg Protein min)					
		Total Proteases	Trypsin	Chymotrypsin	Elastase	Metallo-Protease	Cysteine Protease
Leupeptin ^A^ PMSF ^B^ TLCK ^C^ TPCK ^D^ EDTA ^E^ Iodoacetic acid ^F^	control	25.83 ± 0.45	4.10 ± 0.11	7.35 ± 0.02	8.23 ± 0.02	1.15	0.59 ± 0.05
	0.01	0.12 ± 0.05	3.90 ± 0.11	6.97 ± 0.03	ND	ND	0.28 ± 0.04
	0.05	0.08 ± 0.05	3.82 ± 0.1	6.40 ± 0.02	ND	ND	0.24 ± 0.05
	0.1	0.07 ± 0.05	3.57 ± 0.1	6.18 ± 0.02	6.46 ± 0.01	0.89 ± 0.11	0.19 ± 0.04
	1	0.01 ± 0.05	3.41 ± 0.09	5.28 ± 0.02	5.26 ± 0.03	0.80 ± 0.11	0.16 ± 0.05
	10	ND	3.08 ± 0.1	4.87 ± 0.02	4.18 ± 0.03	0.68 ± 0.11	ND
	50	ND	2.84 ± 0.09	3.64 ± 0.03	3.98 ± 0.0	0.59 ± 0.1	ND
	100	ND	2.12 ± 0.1	2.02 ± 0.02	ND	0.42 ± 0.1	ND

(A) Inhibitor of general proteinase; (B) a general serine proteinase inhibitor (Elastase); (C) inhibitor of Trypsin; (D) TPCK as a chymotrypsin inhibitor; (E) an inhibitor of metalloprotease; (F) an inhibitor of cysteine protease; data are expressed as mean ± SE based on three replicates per tested concentration; ND: Not tested.

## Data Availability

Not applicable.

## Supplementary Materials

The following are available online at https://www.mdpi.com/article/10.3390/insects12050405/s1, Figure S1: GC-MS Chromatogram of (1) *T. vulgaris* and (2) *O. basilicum* cell suspension extracts after 40 days, (1a) p-cymene, (1b) γ-terpinene, (1c) linalool, (1d) β-caryophyllene, (1f) thymol, (2a) β-terpineol, (2b) (*E*)-β-ocimene, (2c) 1,8-cineole, (2d) Estragole, (2f) eugenol and (2g) germacrene D, Table S1: The weight of the callus and the cell suspension produced from *T. vulgaris* and *O. basilicum* at different times of germination in artificial solid MS and liquid LS media with and without infection by *V. dahliae*.

## References

[1] El Hadrami A., Al-Khayri J.M. (2012). Socioeconomic and traditional importance of date palm. Emir. J. Food Agric..

[2] Dembilio Ó., Jaques J.A. (2015). Biology and management of red palm weevil. Sustainable Pest Management in Date Palm: Current Status and Emerging Challenges.

[3] El-Far A., Shaheen H., Abdel-Daim M., Al Jaouni S., Mousa S. (2016). Date palm (*Phoenix dactylifera*): Protection and remedy food. Curr. Trends Nutraceuticals.

[4] Hussain A., Rizwan-ul-Haq M., Al-Jabr A.M., Al-Ayied H.Y. (2013). Managing invasive populations of red palm weevil: A worldwide perspective. J. Food Agric. Environ..

[5] Al-Saqer S.M., Hassan G.M. (2011). Artificial neural networks based red palm weevil (*Rynchophorus ferrugineous*, Olivier) recognition system. Am. J. Agric. Biol. Sci..

[6] Al-Nujiban A.A., Aldosari S.A., Al Suhaibani A.M., Abdel-Azim M.M., Ibrahim S.M.M., Shukla P. (2015). Effect of date palm cultivar on fecundity and development of *Rhynchophorus ferrugineus*. Bull. Insectol..

[7] Milosavljević I., El-Shafie H.A., Faleiro J.R., Hoddle C.D., Lewis M., Hoddle M.S. (2019). Palmageddon: The wasting of ornamental palms by invasive palm weevils, *Rhynchophorus* spp.. J. Pest. Sci..

[8] Shehawy A.A., Ibrahim M.T., Aboutaleb E.S., Qari S.H. (2020). Bioactivity and biochemical efficacy of chitinase and *Justicia brandegeana* extract against Red Palm Weevil *Rhynchophorus ferrugineus* Olivier (Coleoptera: Curculionidae). Food Sci. Nutr..

[9] Idris A.M., Miller T.A., Durvasula R., Fedoroff N. (2015). Bridging the knowledge gaps for development of basic components of Red palm weevil IPM. Sustainable Pest Management in Date Palm: Current Status and Emerging Challenges.

[10] Faleiro J. (2006). A review of the issues and management of the red palm weevil *Rhynchophorus ferrugineus* (Coleoptera: Rhynchophoridae) in coconut and date palm during the last one hundred years. Int. J. Trop. Insect Sci..

[11] Downer A.J., Uchida J.Y., Hodel D.R., Elliott M.L. (2009). Lethal palm diseases common in the United States. HortTechnology.

[12] Kontodimas D., Soroker V., Pontikakos C., Suma P., Beaudoin-Ollivier L., Karamaouna F., Riolo P. (2016). Visual identification and characterization of *Rhynchophorus ferrugineus* and *Paysandisia archon* infestation. Handb. Major Palm Pests Biol. Manag..

[13] Wattanapongsiri A. (1966). A Revision of the Genera *Rhynchophorus* and *Dynamis* (Coleoptera: Curculionidae). Ph.D. Thesis.

[14] Žďárek J., Howard F.W., Moore D., Giblin-Davis R.M., Abad R.G. (2002). Insects on Palms. (Ecological Studies 142.). Biol. Plant..

[15] Fiaboe K., Peterson A.T., Kairo M., Roda A. (2012). Predicting the potential worldwide distribution of the red palm weevil *Rhynchophorus ferrugineus* (Olivier) (Coleoptera: Curculionidae) using ecological niche modeling. Fla. Entomol..

[16] Rugman-Jones P.F., Hoddle C.D., Hoddle M.S., Stouthamer R. (2013). The lesser of two weevils: Molecular-genetics of pest palm weevil populations confirm *Rhynchophorus vulneratus* (Panzer 1798) as a valid species distinct from R. ferrugineus (Olivier 1790), and reveal the global extent of both. PLoS ONE.

[17] Hoddle M., Hoddle C. (2017). Palmageddon: The invasion of California by the South American palm weevil is underway. CAPCA Advis..

[18] Ahmed F., Hussein K., Gad M. (2015). Biological activity of four plant oils, against the red palm weevil, *Rhynchophorus ferrugineus* (Oliver),(Coleoptera: Curculionidae). J. Biosci. Appl. Res..

[19] Abdel-Raheem M., ALghamdi H.A., Reyad N.F. (2020). Nano essential oils against the red palm weevil, *Rhynchophorus ferrugineus* Olivier (Coleoptera: Curculionidae). Entomol. Res..

[20] Cangelosi B., Clematis F., Monroy F., Roversi P.F., Troiano R., Curir P., Lanzotti V. (2015). Filiferol, a chalconoid analogue from *Washingtonia filifera* possibly involved in the defence against the Red Palm Weevil *Rhynchophorus ferrugineus* Olivier. Phytochemistry.

[21] Orfali R., Binsuwaileh A., Al-Ala’a H.A., Bane-Gamea S., Zaidan N., Abdelazim M., Ismael M.A., Perveen S., Majrashi N., Alluhayb K. (2020). Production of a biopesticide on host and Non-Host serine protease inhibitors for red palm weevil in palm trees. Saudi J. Biol. Sci..

[22] Rodríguez-Sifuentes L., Marszalekfer J.E., Chuck-Hernández C., Serna-Saldívar S.O. (2020). Legumes Protease Inhibitors as Biopesticides and Their Defense Mechanisms against Biotic Factors. Int. J. Mol. Sci..

[23] Saad M.M., Gouda N.A., Abdelgaleil S.A. (2019). Bioherbicidal activity of terpenes and phenylpropenes against *Echinochloa crus-galli*. J. Environ. Sci. Health Part B.

[24] Guarino S., Colazza S., Peri E., Bue P.L., Germanà M.P., Kuznetsova T., Gindin G., Soroker V. (2015). Behaviour-modifying compounds for management of the red palm weevil (*Rhynchophorus ferrugineus* Oliver). Pest. Manag. Sci..

[25] Guarino S., Peri E., Bue P.L., Germanà M.P., Colazza S., Anshelevich L., Ravid U., Soroker V. (2013). Assessment of synthetic chemicals for disruption of *Rhynchophorus ferrugineus* response to attractant-baited traps in an urban environment. Phytoparasitica.

[26] AlJabr A.M., Hussain A., Rizwan-ul-Haq M., Al-Ayedh H. (2017). Toxicity of plant secondary metabolites modulating detoxification genes expression for natural red palm weevil pesticide development. Molecules.

[27] Hussain A., Rizwan-Ul-Haq M., AlJabr A.M., Al-Ayedh H. (2019). Lethality of sesquiterpenes reprogramming red palm weevil detoxification mechanism for natural novel biopesticide development. Molecules.

[28] Elansary H.O., Szopa A., Kubica P., Ekiert H., El-Ansary D.O., Al-Mana F.A., Mahmoud E.A. (2020). Saudi *Rosmarinus officinalis* and *Ocimum basilicum* L. Polyphenols and Biological Activities. Processes.

[29] Wojdyło A., Oszmiański J., Czemerys R. (2007). Antioxidant activity and phenolic compounds in 32 selected herbs. Food Chem..

[30] Albohy A., Zahran E.M., Abdelmohsen U.R., Salem M.A., Al-Warhi T., Al-Sanea M.M., Abelyan N., Khalil H.E., Desoukey S.Y., Fouad M.A. (2020). Multitarget in silico studies of *Ocimum menthiifolium*, family Lamiaceae against SARS-CoV-2 supported by molecular dynamics simulation. J. Biomol. Struct. Dyn..

[31] Zahran E.M., Abdelmohsen U.R., Kolkeila A., Salem M.A., Khalil H.E., Desoukey S.Y., Fouad M.A., Kamel M.S. (2020). Anti-epileptic potential, metabolic profiling and in silico studies of the aqueous fraction from *Ocimum menthiifolium* benth, family Lamiaceae. Nat. Prod. Res..

[32] Zahran E.M., Abdelmohsen U.R., Khalil H.E., Desoukey S.Y., Fouad M.A., Kamel M.S. (2020). Diversity, phytochemical and medicinal potential of the genus *Ocimum* L. (Lamiaceae). Phytochem. Rev..

[33] Zahran E.M., Abdelmohsen U.R., Ayoub A.T., Salem M.A., Khalil H.E., Desoukey S.Y., Fouad M.A., Kamel M.S. (2020). Metabolic profiling, histopathological anti-ulcer study, molecular docking and molecular dynamics of ursolic acid isolated from *Ocimum forskolei* Benth. (family Lamiaceae). S. Afr. J. Bot..

[34] Al-Asmari A.K., Athar M.T., Al-Faraidy A.A., Almuhaiza M.S. (2017). Chemical composition of essential oil of *Thymus vulgaris* collected from Saudi Arabian market. Asian Pac. J. Trop. Biomed..

[35] Chenni M., El Abed D., Rakotomanomana N., Fernandez X., Chemat F. (2016). Comparative study of essential oils extracted from Egyptian basil leaves (*Ocimum basilicum* L.) using hydro-distillation and solvent-free microwave extraction. Molecules.

[36] Padalia R., Verma R., Chauhan A., Chanotiya C. (2013). Changes in aroma profiles of 11 Indian *Ocimum taxa* during plant ontogeny. Acta Physiol. Plant..

[37] Murthy H.N., Lee E.-J., Paek K.-Y. (2014). Production of secondary metabolites from cell and organ cultures: Strategies and approaches for biomass improvement and metabolite accumulation. Plant. Cell Tissue Organ. Cult. (PCTOC).

[38] Açıkgöz M.A. (2020). Establishment of cell suspension cultures of *Ocimum basilicum* L. and enhanced production of pharmaceutical active ingredients. Ind. Crops Prod..

[39] Waterman P.G., Mole S. (1994). Analysis of Phenolic Plant Metabolites.

[40] Shukla P., Vidyasagar P., Aldosari S.A., Abdel-Azim M. (2012). Antifeedant activity of three essential oils against the red palm weevil, *Rhynchophorus ferrugineus*. Bull. Insectol..

[41] Lowry O., Rosebrough N.J., Farr A., Randall R.J. (1951). Protein Measurement with the Folin Phenol Reagent. J. Biol. Chem..

[42] Olga L., Ibrahim M., Candas N., Koller N., Bauer L., Bulla L. (2002). Changes in proteases activity and cry 3Aa toxin binding in the Colorado potato beetle: Implications for insect resistance to *Bacillus thuringiensis* toxins. Insect Biochem. Mol. Biol..

[43] Finney D.J. (1971). Probit Analysis.

[44] Cvikrova M., Hrubcova M., Eder J., Binarova P. (1996). Changes in the levels of endogenous phenolics, aromatic monoamines, phenylalanine ammonia-lyase, peroxidase and auxin oxidase activities during initiation of alfalfa embryogenic and nonembryogenic calli. Plant. Physiol. Biochem. (Paris).

[45] Ross D.C., Brown T.M. (1982). Inhibition of larval growth in *Spodoptera frugiperda* by sublethal dietary concentrations of insecticides. J. Agric. Food Chem..

[46] Lewis N.G., Yamamoto E. (1990). Lignin: Occurrence, biogenesis and biodegradation. Annu. Rev. Plant Biol..

[47] Bolwell G.P., Robbins M.P., Dixon R.A. (1985). Metabolic changes in elicitor-treated bean cells: Enzymic responses associated with rapid changes in cell wall components. Eur. J. Biochem..

[48] Kefeli V.I., Kalevitch M.V., Borsari B. (2003). Phenolic cycle in plants and environment. J. Cell Mol. Biol..

[49] López Arnaldos T., Muñoz R., Ferrer M.A., Calderón A.A. (2001). Changes in phenol content during strawberry (Fragaria× ananassa, cv. Chandler) callus culture. Physiol. Plant..

[50] Mato M., Rua M., Ferro A. (1988). Changes in levels of peroxidases and phenolics during root formation in Vitis cultured in vitro. Physiol. Plant..

[51] Pickens C.L., Airavaara M., Theberge F., Fanous S., Hope B.T., Shaham Y. (2011). Neurobiology of the incubation of drug craving. Trends Neurosci..

[52] Atanasov A.G., Waltenberger B., Pferschy-Wenzig E.-M., Linder T., Wawrosch C., Uhrin P., Temml V., Wang L., Schwaiger S., Heiss E.H. (2015). Discovery and resupply of pharmacologically active plant-derived natural products: A review. Biotechnol. Adv..

[53] Ochoa-Villarreal M., Howat S., Hong S., Jang M.O., Jin Y.-W., Lee E.-K., Loake G.J. (2016). Plant cell culture strategies for the production of natural products. BMB Rep..

[54] Mathew R., Sankar P.D. (2014). Comparison of major secondary metabolites quantified in elicited cell cultures, non-elicited cell cultures, callus cultures and field grown plants of *Ocimum*. Int. J. Pharm. Pharm. Sci..

[55] Michaud N.R., Fabian J.R., Mathes K.D., Morrison D.K. (1995). 14-3-3 is not essential for Raf-1 function: Identification of Raf-1 proteins that are biologically activated in a 14-3-3-and Ras-independent manner. Mol. Cell. Biol..

[56] Li H., Child M.A., Bogyo M. (2012). Proteases as regulators of pathogenesis: Examples from the Apicomplexa. Biochim. Biophys. Acta (BBA) Proteins Proteom..

