# Supplementary material for: Bio-Insecticide of Thymus vulgaris and Ocimum basilicum Extract from Cell Suspensions and Their Inhibitory Effect against Serine, Cysteine, and Metalloproteinases of the Red Palm Weevil (Rhynchophorus ferrugineus)
Source: Insects. 2021 Apr 30;12(5):405. doi: 10.3390/insects12050405 (PMC8147177; doi:10.3390/insects12050405)
Supplement: Supplementary file 1 [file insects-12-00405-s001.zip › insects-1194671-s-4.30.pdf]

Supplementary materials

Bio-insecticide of *Thymus vulgaris* and *Ocimum basilicum* extract from cell suspensions and their inhibitory effect against serine, cysteine, and metalloproteinases of the Red Palm Weevil (*Rhynchopho-rus ferrugineus*)

Hossam Moustafa Darrag [1, 2, *] Mohammed Refdan Alhajhoj [3], and Hany Ezzat Khalil [4,5]

[1] Research and Training Station, King Faisal University, Al-Ahsa 31982, Saudi Arabia; hdarag@kfu.edu.sa

[2] Pesticide Chemistry and Technology Department, Faculty of Agriculture, Alexandria University, Egypt; hdarag@kfu.edu.sa

[3] Arid Land Agriculture Department, College of agricultural and Food Sciences, King Faisal University, Al-Ahsa 31982, Saudi Arabia; malhajhoj@kfu.edu.sa

[4] Department of Pharmaceutical Sciences, College of Clinical Pharmacy, King Faisal University, Al-Ahsa 31982, Saudi Arabia; heahmed@kfu.edu.sa

[5] Department of Pharmacognosy, Faculty of Pharmacy, Minia University, Minia 61519, Egypt; heahmed@kfu.edu.sa

* Correspondence: hdarag@kfu.edu.sa; Tel.: +966508299027

Table S1. The weight of the callus and the cell suspension produced from *T. vulgaris* and *O. basilicum* at different times of germination in artificial solid MS and liquid LS media with and without infection by *V. dahliae*.

**Age of Callus (days)**	**Weight of Callus (g)/Petri dish ± SD**			
	T. vulgaris		***O. basilicum***	
	Without Infection	With Infection	Without Infection	With Infection
**5**	2.16^{p}±0.03	2.96^{n}±0.07	2.24^{l}±0.14	3.41^{k}±0.06
**10**	2.87^{o}±0.03	3.23^{m}±0.01	3.29^{k}±0.23	4.29^{j}±0.01
**15**	3.49^{l}±0.03	4.18^{k}±0.04	4.37^{j}±0.02	5.85^{h}±0.08
**20**	4.25^{j}±0.01	4.82^{i}±0.01	5.22^{i}±0.02	6.32^{g}±0.04
**25**	5.17^{h}±0.04	5.87^{g}±0.04	6.17^{g}±0.09	8.31^{d}±0.13
**30**	5.96^{f}±0.06	6.74^{d}±0.04	6.76^{f}±0.10	8.85^{c}±0.10
**35**	6.26^{e}±0.03	7.23^{b}±0.01	7.27^{e}±0.01	9.83^{b}±0.04
**40**	7.11^{c}±0.01	7.80^{a}±0.02	8.25^{d}±0.10	10.51^{a}±0.06
F	7515		2028	
Df	15,32		15,32	
**Sample (at intervals in days)**	**Weight of Cell Suspension (g)/200 mL media ± SD**			
	T. vulgaris		***O. basilicum***	
	Without Infection	With Infection	Without Infection	With Infection
**5**	1.45^{m}±0.04	2.32^{k}±0.01	1.91^{o}±0.07	2.46^{m}±0.06
**10**	1.92^{l}±0.02	2.99^{i}±0.02	2.26^{n}±0.05	3.15^{l}±0.04
**15**	2.37^{j}±0.03	3.14^{h}±0.05	3.27^{k}±0.04	4.28^{h}±0.04
**20**	3.11^{h}±0.01	3.94^{g}±0.04	3.36^{j}±0.03	4.86^{g}±0.04
**25**	3.95^{g}±0.03	4.55^{d}±0.01	4.14^{i}±0.07	5.21^{f}±0.02
**30**	4.23^{f}±0.03	5.00^{c}±0.01	5.19^{f}±0.05	6.34^{c}±0.06
**35**	4.43^{e}±0.01	5.26^{b}±0.04	5.62^{e}±0.04	7.03^{b}±0.07
**40**	5.03^{c}±0.02	5.35^{a}±0.04	6.24^{d}±0.11	7.42^{a}±0.05
F	5760		2700	
**Df**	15,32		15,32	

Data are averages ± SD of three replicates. Values followed by the same letter within a column are not significantly different (P ≤ 0.0001 ≡ V.H.Sig. for all values) according to the Student–Newman–Keuls (SNK) test. F: F ratio, df: degree of freedom, P: p-value (significance of the F ratio).

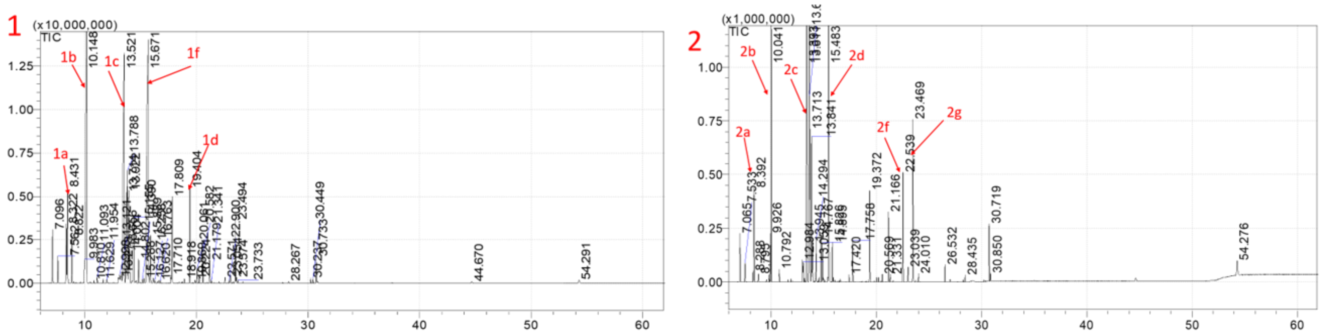

Figure S1. GC-MS Chromatogram of (1) *T. vulgaris* and (2) *O. basilicum* cell suspension extracts after 40 days, (1a) p-cymene, (1b) γ-terpinene, (1c) linalool, (1d) β-caryophyllene, (1f) thymol, (2a) β-terpineol, (2b) (*E*)-β-ocimene, (2c) 1,8-cineole, (2d) Estragole, (2f) eugenol and (2g) germacrene D
